# Supplementary material for: Does Total Neoadjuvant Therapy Impact Surgical Precision in Total Mesorectal Excision? A Nationwide Survey of the Experiences of Expert Surgeons
Source: Cancers (Basel). 2025 Jan 17;17(2):283. doi: 10.3390/cancers17020283 (PMC11763819; doi:10.3390/cancers17020283)
Supplement: Supplementary file 1 [file cancers-17-00283-s001.zip › cancers-3309079-supplementary.pdf]

# TNT Rectal Cancer Survey (Tarkan Jäger et al. 2024)

## *Does Total Neoadjuvant Therapy Impacts Surgical Precision in Total Mesorectal Excision? A Nationwide Survey on Expert Experiences*

## 1. General

**Questions:**

1. **Tumor Board Presentation**
    - Question: Are patients with primary rectal cancer routinely presented at a multidisciplinary tumor board in your hospital?
    - Choices: Yes, No
2. **Annual Presentation of Cases**
    - Question: How many primary rectal cancer cases are presented in your hospital per year?
    - Choices: 0-20, 21-40, 41-60, 61-80, 81-100, >100 cases
3. **Watch and Wait Protocol**
    - Question: Do you apply a Watch and Wait protocol for patients with clinically complete response to neoadjuvant therapy in your hospital?
    - Choices: Yes, No
4. **MERCURY Grading**
    - Question: Is MERCURY grading routinely determined in your hospital?
    - Choices: Yes, No
5. **Neoadjuvant Therapy Concepts**
    - Question: Which neoadjuvant therapy concepts for patients with advanced rectal cancer are offered in your hospital?
    - Choices: Short-term radiotherapy, Chemotherapy alone, Chemoradiotherapy, Total neoadjuvant therapy (TNT), None of the above
6. **Availability of TNT**
    - Question: Is total neoadjuvant therapy (TNT) offered to patients with rectal cancer in your hospital?
    - Choices: Yes, No
7. **TNT Protocol**
    - Question: Which protocol is used for TNT in your hospital?
    - Choices: Induction (chemotherapy first), Consolidation (radiation first), Different, Other
    - Conditional Text Field (if 'Other' is selected): Please enter the protocol here

## 2. Rectal Resection after Classical Neoadjuvant Therapy (CNT)

**Questions:**

1. **Experience with CNT**
    - Question: Have you operated on patients after classical neoadjuvant therapy (CNT) (e.g. short-course, long-course radiotherapy)?
    - Choices: Yes, No
2. **Timing of Surgery Post-CNT**
    - Question: When do you generally operate on patients with residual tumor after CNT?

    - Choices: Immediately after completion of short-term radiotherapy, 6-7 weeks after CNT, 8-10 weeks after CNT, 11-12 weeks after CNT, 13-14 weeks after CNT, > 14 weeks after CNT, Depending on response earlier or later
3. **Preferred Surgical Technique Post-CNT**
    - Question: Which surgical technique did you prefer for your last CNT patients?
    - Choices: Robot-assisted, Transanal, Open, Laparoscopic, Other
    - Conditional Text Field (if 'Other' is selected): Please enter your surgical technique here
4. **Routine Protective Stoma Post-CNT**
    - Question: Do you routinely create a protective stoma after CNT?
    - Choices: Yes, No
5. **Preferred Type of Protective Stoma Post-CNT**
    - Question: Which protective stoma do you prefer to create after CNT?
    - Choices: Ileostomy, Transversostomy
6. **Factors Influencing Stoma Creation Post-CNT**
    - Question: Please specify what the creation of a protective stoma after CNT depends on?
    - Choices: Tumor height, Previous therapy, Other reasons

## 3. Rectal Resection after Total Neoadjuvant Therapy (TNT)

**Questions:**

1. **Experience with TNT**
    - Question: Have you operated on patients after total neoadjuvant therapy (TNT)?
    - Choices: Yes, No
2. **Timing of Surgery Post-TNT**
    - Question: When do you generally operate on patients with residual tumor after TNT?
    - Choices: Immediately after completion of short-term radiotherapy, 6-7 weeks after TNT, 8-10 weeks after TNT, 11-12 weeks after TNT, 13-14 weeks after TNT, > 14 weeks after TNT, Depending on response earlier or later
3. **Preferred Surgical Technique Post-TNT**
    - Question: Which surgical technique did you prefer for your last TNT patients?
    - Choices: Robot-assisted, Transanal, Open, Laparoscopic, Other
    - Conditional Text Entry: Please enter your surgical technique here (if 'Other' is selected)
4. **Routine Protective Stoma Post-TNT**
    - Question: Do you routinely create a protective stoma after TNT?
    - Choices: Yes, No
5. **Preferred Type of Protective Stoma Post-TNT**
    - Question: Which protective stoma do you prefer to create after TNT?
    - Choices: Ileostomy, Transversostomy
6. **Factors Influencing Stoma Creation Post-TNT**
    - Question: Please specify what the creation of a protective stoma after TNT depends on?
    - Choices: Tumor height, Previous therapy, Other reasons
7. **Experience with Regrowth after TNT**
    - Question: Have you operated on patients with regrowth after clinically complete response (cCR) following TNT?

- Choices: Yes, No

8. **Timing of Surgery after Watch and Wait Protocol Post-TNT**
    - Question: What was the average time of surgery after Watch and Wait protocol following TNT?
    - Choices: < 6 months after cCR, between 6 and 12 months after cCR, between 12 and 24 months after cCR, > 24 months after cCR

## 4. Personal Experiences during Rectal Resection Post-TNT: Scenario 1

**Questions:**

1. **Preparation Experience during TME Post-TNT**
    - Sub-questions:
        - A. No difference
        - B. Difficulty in layer identification
        - C. Increased bleeding
        - D. Fragile tissue, tears easily
    - Choices: 1. Strongly disagree, 2. Disagree, 3. Neutral, 4. Agree, 5. Strongly agree
2. **Changes in TME Quality Post-TNT (MERCURY I-III)**
    - Sub-questions:
        - A. No, similar quality to CNT
        - B. Yes, better quality than CNT
        - C. Yes, worse quality than CNT
    - Choices: 1. Strongly disagree, 2. Disagree, 3. Neutral, 4. Agree, 5. Strongly agree
3. **Anastomotic Leakage Experience Post-TNT**
    - Sub-questions:
        - A. No abnormalities
        - B. Increased anastomotic insufficiencies
    - Choices: 1. Strongly disagree, 2. Disagree, 3. Neutral, 4. Agree, 5. Strongly agree
4. **Wound Healing Issues Post-Abdominoperineal Excision Post-TNT**
    - Sub-question:
        - A. Observed increased wound healing issues
    - Choices: 1. Strongly disagree, 2. Disagree, 3. Neutral, 4. Agree, 5. Strongly agree

## 5. Personal Experiences during Rectal Resection Post-TNT: Scenario 2

**Questions:**

1. **Preparation Experience during TME Post-Regrowth under Watch and Wait Strategy**
    - Sub-questions:
        - A. No difference
        - B. Difficulty in layer identification
        - C. Increased bleeding
        - D. Fragile tissue, tears easily
    - Choices: 1. Strongly disagree, 2. Disagree, 3. Neutral, 4. Agree, 5. Strongly agree

2. **Changes in TME Quality Post-Regrowth (MERCURY I-III)**
   - Sub-questions:
     - A. No, similar quality to CNT
     - B. Yes, better quality than CNT
     - C. Yes, worse quality than CNT
   - Choices: 1. Strongly disagree, 2. Disagree, 3. Neutral, 4. Agree, 5. Strongly agree
3. **Anastomotic Leakage Experience Post-Regrowth**
   - Sub-questions:
     - A. No abnormalities
     - B. Increased anastomotic insufficiencies
   - Choices: 1. Strongly disagree, 2. Disagree, 3. Neutral, 4. Agree, 5. Strongly agree
4. **Wound Healing Issues Post-Abdominoperineal Excision Post-Regrowth**
   - Sub-question:
     - A. Observed increased wound healing issues
   - Choices: 1. Strongly disagree, 2. Disagree, 3. Neutral, 4. Agree, 5. Strongly agree

## 6. Personal Data

**Questions:**

1. **Personal Data**
   - Question: Do you want to personalize your responses?
   - Choices: Yes, No
   - Conditional Text Entry: Name, Affiliation, Email Address (if 'Yes' is selected)
2. **Further Topic Development**
   - Question: Should this topic be further developed (e.g. nationwide ACO-ASSO Register on TNT, Watch and Wait)?
   - Choices: 1. Strongly disagree, 2. Disagree, 3. Neutral, 4. Agree, 5. Strongly agree
3. **Additional Comments**
   - Question: Do you have any additional comments?
   - Choices: Free text area for comments
